# Recrystallization Mechanisms of Aluminum and Aluminum Oxide Interfaces through Reactive Simulations

**DOI:** 10.1021/jacsau.5c01074

**Published:** 2025-09-10

**Authors:** Hao Zhao, Fernando Bresme

**Affiliations:** † Department of Chemistry, Molecular Sciences Research Hub, 4615 Imperial College, London W12 0BZ, U.K.; ‡ State Key Laboratory of Multiphase Flow in Power Engineering, Xi’an Jiaotong University, Xi’an, Shaanxi 710049, China

**Keywords:** metal oxides, aluminum, crystallization mechanism, ReaxFF molecular dynamics, unsupervised clustering algorithm

## Abstract

Aluminum and alumina are essential materials used in various energy processes and devices. In this study, we conduct an atomic-level investigation into the microscopic mechanisms that govern the recrystallization (crystal growth from its melt) of aluminum and aluminum oxide interfaces. We utilize a reactive force field (ReaxFF) along with bond-orientational order parameters and unsupervised clustering algorithms to clarify the barrierless nature of the ultrafast metallic growth processes of aluminum. Our analysis provides valuable insights into the microscopic mechanisms that facilitate the incorporation of atoms into alumina, which is a crucial step in the crystal growth of this metal oxide. For the crystal growth of alumina we identify a sequential process where oxygen atoms first occupy lattice sites before aluminum atoms are integrated. This mechanism involves energy barriers that may explain the slow crystal growth rates reported in the crystallization of aluminum oxide. Furthermore, we report a significant correlation between the incorporation of oxygen into the crystal structure and the modification of the atomic charge. These findings enhance our understanding of the distinct crystallization behaviors of metals and metal oxide interfaces, offering microscopic insights for developing materials with improved performance characteristics.

## Graphical abstract

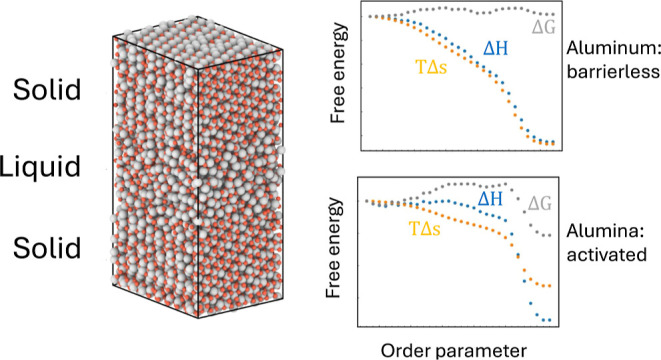

## Introduction

Aluminum exists in two primary forms: metallic aluminum and aluminum oxide (alumina). When crystallizing from melts, pure metals exhibit ultrafast recrystallization (growth) rates from the melt ∼102 m/s.^1,2^ In contrast, the growth rates of metal oxides generally vary from 10^–9^ to 10^–3^ m/s.^3^ The slow crystallization process of alumina greatly affects its phase composition, interfacial structure, microstructure and, ultimately, its thermal and mechanical properties, especially during the transition from θ-Al_2_O_3_ to α-Al_2_O_3_ above 1200 °C.^4^

The rapid crystallization of aluminum has been linked to a barrierless crystal growth kinetics process.^2^ However, the primary free energy pathways for atomic movement between the liquid and solid phases have yet to be quantified at the microscopic level. Similarly, the microscopic processes and energy pathways through which aluminum and oxygen atoms integrate into alumina crystals remain unresolved. Understanding these mechanisms is crucial for developing materials with enhanced performance characteristics. Such enhancements include improved thermal conductivity for better heat dissipation in electronics,^5^ increased corrosion resistance,^6^ and optimized optical properties for applications in energy-efficient systems.^7^ All of these properties are influenced by the crystallization of aluminum oxide.

To investigate crystallization mechanisms, it is essential to study crystal growth at the atomic scale. The reactive force field (ReaxFF),^8,9^ parametrized using ab initio computations, is widely used in classical simulations of complex systems. This force field allows for the investigation of chemical reactivity, including modeling charge transfer processes. ReaxFF dynamically defines chemical bonds based on interatomic distances and atom types, enabling the modeling of bond-breaking and bond-forming events.

As a result, ReaxFF is extensively used to study the dynamic behavior and reactivity of aluminum-based compounds in various environments. Examples include the oxidation of aluminum,^10,11^ carbon coating processes on aluminum nanoparticles,^12^ and the adsorption of water on alumina.^13,14^ However, the dynamics of crystal growth in the ReaxFF framework has not been considered before.

ReaxFF offers unique advantage for our study by (1) dynamically modeling bond-breaking/forming events; (2) computing atomic charges via the Electronegativity Equalization Method, capturing polarization and potentially charge transfer effects inaccessible to more traditional force fields, (3) providing nanosecond-scale simulations while maintaining atomic resolution and high chemical accuracy, bridging ab initio parametrized force fields and experiments. Furthermore, ReaxFF provides a unified framework to investigate aluminum and alumina, allowing direct comparison of their crystallization mechanisms.

Quantifying the energy pathway associated with crystal formation is essential in understanding reaction kinetics. We can analyze these free energy pathways by measuring the Gibbs free energy along a “reaction” coordinate while maintaining constant pressure and temperature. Computer simulations provide a detailed microscopic approach to calculating the enthalpic contributions of individual atoms to the Gibbs free energy. This is achieved by evaluating interatomic potential energies, pressure, and atomic volumes, which helps us understand how atoms progress along the reaction coordinate as they integrate into a growing crystal. However, calculating the atomic entropic contribution is more complex and typically requires thermodynamic integration methods that involve the entire system of interest.

Recently, Piaggi and Parrinello^15^ introduced a per-atom configurational entropy to differentiate between solid and liquid phases. This definition allows for calculating the entropy of individual atoms, enabling the distinction between various crystalline structures, grain boundaries, and solid–liquid interfaces. Here, we build upon this method to calculate the entropic contribution of individual atoms. We use this entropic contribution to compute atomic Gibbs free energies. We employ this approach to investigate the crystal growth process of aluminum and alumina under supercooled conditions using the ReaxFF method and in nanosecond time scale. The Steinhardt order parameter, *q*_6_^′^, defines the “reaction” coordinate, allowing us to monitor changes in atomic properties during the crystallization process. These changes are quantified by calculating the potential energy, enthalpy, entropy, and free energy for each atom.

Our analysis combines the order parameter with local entropy, atomic energies, atomic volumes, and ultimately the per-atom Gibbs free energy. We examine thousands of molecular simulation trajectories using unsupervised clustering algorithms to identify the microscopic pathways that lead to recrystallization events from the melt. This investigation provides a detailed understanding of the barrierless nature of aluminum crystallization at the microscopic level. Additionally, our methodology, which has been validated on silicon systems, demonstrates growth rates that are consistent with analyses from previous literature.

The barrierless nature of recrystallization in aluminum contrasts with the more complex free energy landscape associated with alumina crystallization. To quantify the free energy barriers in alumina, we conducted simulations in the gigapascal pressure regime. It has been shown that high pressure facilitates the in situ observation of recrystallization on the nanosecond time scale targeted by molecular simulations. High pressure conditions (0.1 GPa and higher) are relevant in materials science,^16,17^ geophysics of the mantle.^18–20^ We provide evidence of a process controlled by an energy barrier, where oxygen and aluminum atoms are sequentially incorporated into preformed alumina crystal faces. Furthermore, we demonstrate that the activation energies for the crystallization process increase as pressure decreases leading to a reduction of the recrystallization speed.

## Methods

### ReaxFF Force Field

The ReaxFF interatomic potential^8,9^ is defined as

1 $$E_{total}=E_{bond}+E_{over}+E_{lp}+E_{val}+E_{tors}+E_{vdW}+E_{Coulomb}$$

where *E*_total_ denotes the total energy of the system, which comprises bond formation energy, *E*_bond_, the over/under coordination energy, *E*_over_, the lone pair energy, *E*_lp_, the valence angle energy, *E*_val_, the torsion energy, *E*_tors_, the van der Waals energy, *E*_vdW_, and the Coulombic energy, *E*_Coulomb_. ReaxFF uses the bond order (BO) to compute and describe the chemical bond interactions, with BO being a function of atom distances and types. More details are available in refs 8 and 21 . In this work, the ReaxFF parameter set employed was the Al/O-2015 set, optimized by Hong in ref 22 using the one-parameter parabolic extrapolation against DFT training sets.^23,24^ The first Al/O parameter set was developed by Zhang et al.^25^ during their study of the Al/α-Al_2_O_3_ interface. Subsequently, Hong and van Duin^22^ revised the Al/O parameter set (Al/O-2015) and validated its accuracy in the study of aluminum oxidation.

### Simulation Configuration

We employed a solid/liquid interface to simulate the crystallization/melting process. The interface was prepared by merging separately relaxed supercooled liquids and solids at the same specified temperature. The supercooled liquids and solids were obtained from single-phase bulk heating and cooling processes. The initial configurations for bulk aluminum and alumina were constructed using crystallography data.^26,27^ Pymatgen^28^ was used to convert the trigonal alumina structure to a cubic cell structure. Supercell operations were employed to create a 32 × 32 × 32 Å^3^ simulation box with 2048 aluminum atoms in the [100] direction, and a 29 × 29 × 40 Å^3^ simulation box with 1920 aluminum atoms in the [111] direction. A 33 × 33 × 39 Å^3^ box with 2016 aluminum and 3024 oxygen atoms was constructed to simulate alumina. The simulation box dimensions were determined by replicating the unit cell along each axis until the target size was reached; when decimal values arose, they were rounded to the nearest integer multiple of the unit cell parameter to ensure compatibility with periodic boundary conditions, which were applied in all three dimensions. A schematic illustration of the simulation cell construction can be found in our previous work.^29^ The selected cross-sectional areas ensure the system is large enough to avoid size effects while maintaining reasonable computational costs. The size of the box in the longitudinal dimension, ∼80 Å, chosen for the explicit interface simulations, is sufficiently long to minimize interface–interface correlations and to track the crystal growth dynamics over a sufficiently long time period.

The initial speeds were set using a Gaussian distribution to target 400 and 1000 K, respectively, approximately half of their experimental melting points. The solid was heated to 1200 K for aluminum and 3000 K for alumina, then cooled to 400 K for aluminum and 1000 K for alumina. Direct coexistence simulations were performed for 1 ns using the *NPT* ensemble. More details on the preparation of the single-phase simulations, thermostat and barostat details, and temperature control are included in the Supporting Information. Figure S1 in Supporting Information summarizes the simulation workflow employed in this work.

In ReaxFF, atomic charges were computed at each time step using the Electronegativity Equalization Method (EEM),^30^ which assigns charges based on predefined electronegativity and hardness parameters. While these charges are not equivalent to ab initio-derived quantum mechanical charges, they remain valuable for identifying qualitative trends in polarization and charge transfer trends.

For aluminum, the simulations were conducted at a pressure of 1 atm. In contrast, simulations were performed under high pressures of in the range 5–20 GPa for alumina to accelerate the crystallization process and investigate the impact of pressure on the recrystallization dynamics. We demonstrated in our previous work that complete crystallization, within the nanosecond time scales, can be achieved at high pressure.^29^ Similar observations have been reported in simulations of silica system.^31^

### Calculation of Per-Atom Gibbs Free Energies

Before introducing the per-atom Gibbs free energy, we defined a per-atom reaction coordinate based on the order parameter *q*_6_^′^.^32,33^ Unlike the time-averaged Lindemann index,^34^ the order parameter *q*_6_^′^, is a distance-based, weighted Steinhardt order parameter function, which quantifies the atomic ordering and can instantaneously distinguish whether an atom is in a liquid or solid phase. The order parameter is defined as

2 $$q_{6}^{\prime }\left(i\right)=\sqrt{\frac{4\pi }{13}\sum_{m=-6}^{6}{\left|\sum_{f\in F\left(i\right)}\frac{A\left(f\right)}{A_{i}}Y_{6m}\left(\theta_{f},\varphi_{f}\right)\right|}^{2}}$$

Here, *Y*_6m_ is the spherical harmonic with the components of degree 6, *A*(*f*) is the surface area of the facet *f*, for an atomic Voronoi cell, where the *f* belongs to the Voronoi cell boundary F(i) of atom *i*, and $A_{i}=\sum_{f\in F\left(i\right)}A\left(f\right)$ . The Voronoi tessellation was calculated using the Voro++ library.^35^ A *q*_6_^′^ below 0.2 indicates a disordered liquid state, while above 0.4 indicates an ordered solid state.

The per-atom Gibbs free energy is defined using the following equation: *g* = *h* – *Ts* = *p*_e_ + *k*_e_ + *pv* – *Ts*, where *p*_e_ is the potential energy, *p* is the pressure, *v* is the per-atom volume, *k*_e_ is the kinetic energy, *T* is the temperature, and *s* is the entropy. *p*_e_ can be directly obtained from the calculation of the interatomic interactions. During the phase change, we consider the average temperature of the system, *T*, and correspondingly the kinetic energy *k*_e_ = 3*k*_B_ *T*/2. The per-atom volume was calculated using the Voronoi tessellation method. All these properties can be easily obtained during an MD simulation. However, calculating the entropy per atom is more difficult. We estimated this entropy using the two-body excess entropy method,^15,36–38^ where the two-body excess entropy is defined as

3 $$s\approx s_{2}=-2\pi \rho k_{B}\int_{0}^{r_{m}}\left[g\left(r\right)\ln g\left(r\right)-g\left(r\right)+1\right]r^{2}\;dr$$

where ρ represents the density of the system, *g*(*r*) is the radial distribution function, and *r*_m_ is an upper limit of integration.

Piaggi and Parrinello^15^ proposed using a per-atom *s*_2_ to differentiate between solid and liquid phases. The entropy of individual atoms can also effectively distinguish between different crystal structures and grain boundaries. After precise calibration, we have expanded this method to quantify the atomic entropy entering the per-atom Gibbs free energy, *g*. Huang and Widom^39^ investigated the accuracy of *s*_2_ in predicting the entropy of Lennard-Jones fluids at various temperatures and densities. They compared *s*_2_ with different series expansions of *n*-body distribution functions. The results demonstrated that *s*_2_ can predict how the entropy varies with temperature.

For the calibration of the per-atom entropy calculation, we used the Gibbs energy difference between the liquid and solid phases in the supercooled regime^40^

4 $$\Delta G=G_{s}-G_{l}\approx \frac{\Delta H_{m}}{T_{m}}\times \left(T-T_{m}\right)$$

We introduce the fitting factor *f*_0_(*T*) for *s*_2_

5 $$f_{0}\left(T\right)=\frac{\Delta H-\Delta G}{T\times \Delta s_{2}}\approx \frac{\Delta H-\Delta H_{m}/T_{m}\times \left(T-T_{m}\right)}{T\cdot \left[s_{2}^{s}\left(T\right)-s_{2}^{l}\left(T\right)\right]}$$

where Δ*H* = *H*_s_ – *H*_l_, and Δ*H*_m_ is the enthalpy of melting, which can be accessed from molecular dynamics simulation at the melting temperature *T*_m_.^29^

By combining the *q*_6_^′^ “reaction” coordinate with the atomic Gibbs free energy, we calculated the free energy associated with the pathway taken by atoms during the crystallization process under supercooled conditions. Our approach offers an efficient description of the per-atom crystallization process using a direct method

6 $$g_{i}\left[q_{6}^{\prime }\left(i\right)\right]=\frac{3}{2}k_{b}T+p_{e}\left(i\right)+pv\left(i\right)-f_{0}\times Ts_{2}\left(i\right)$$

where *g*_*i*_ represents the per-atom free energy as a function of the *q*_6_^′^(*i*) order parameter. The term *p*_e_(*i*) denotes the potential energy of atom *i*, while *pv*(*i*) represents the pressure–volume work. The variable *f*_0_ is an empirical factor, and *f*_0_ × *Ts*_2_(*i*) is the temperature-scaled two-body entropy term.

## Results and Discussion

### The Crystal Growth of Aluminum

The temperature dependence of *f*_0_ from eq 5 is shown in Figure 1. Using the same ReaxFF method outlined in ref.,^29^ we determined the melting point of aluminum to be 858 ± 2 K. Our results indicate that *f*_0_ increases as temperature rises. We employed the *f*_0_(*T*) extracted from the data in Figure 1 to perform our calculations of Al at T = 800 K.

**Figure 1:**
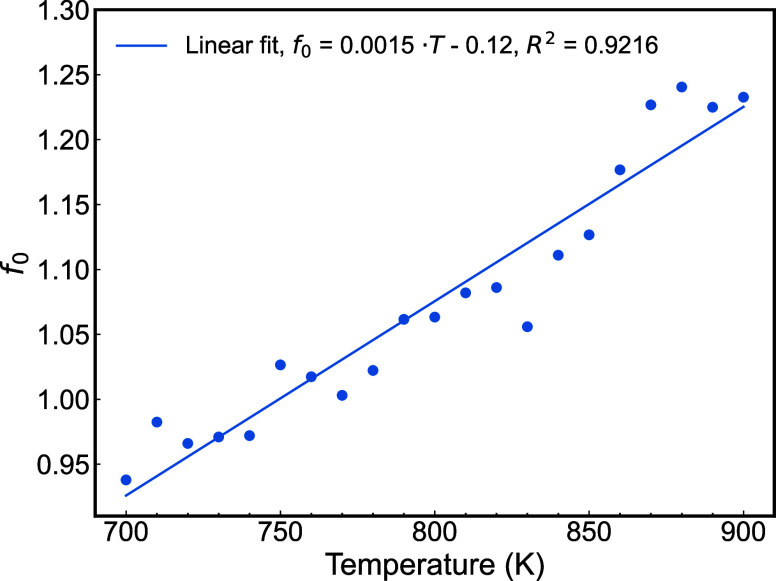
Temperature dependence of the fitting parameter *f*_0_ of aluminum. *f*_0_ was calculated using eq 5 in the main text. We note there is not an ideal linear relationship between temperature and *f*_0_. However, the temperature dependence of *f*_0_ can be modeled well using a linear fit (see data in the inset).

Figure 2 shows the time series evolution of the *q*_6_^′^ order parameter at the solid/liquid aluminum interface, computed at a temperature of 800 K and a pressure of 1 atm. This temperature is 58 K below the melting point, as determined by the ReaxFF method in ref 29. Initially, the solid and liquid phases appear as distinct regions: the solid is represented with red color and the liquid with blue color (*q*_6_^′^ < 0.20). During the crystal growth, the ultrafast interfacial speeds reach values of ∼67 m/s. This speed was calculated by tracking the displacement of the interface, using *q*_6_^′^ = 0.40. The interfacial order parameter is nonuniform since the *q*_6_^′^ is a local structural measure. Also, local thermal fluctuations influence its value (see Figure 2). After 0.03 ns (see Figure 2), the system approaches equilibrium, and the potential energy stabilizes (see Figure S2). As the entire system solidifies, the phase boundaries become less distinct. The *q*_6_^′^ parameter provides an excellent approach to instantaneously classify the state of each atom as either solid or liquid (see Figure S3). Therefore, we used this parameter as the “reaction” coordinate (for crystallization or melting) to represent the free energy during the crystallization process.

**Figure 2:**
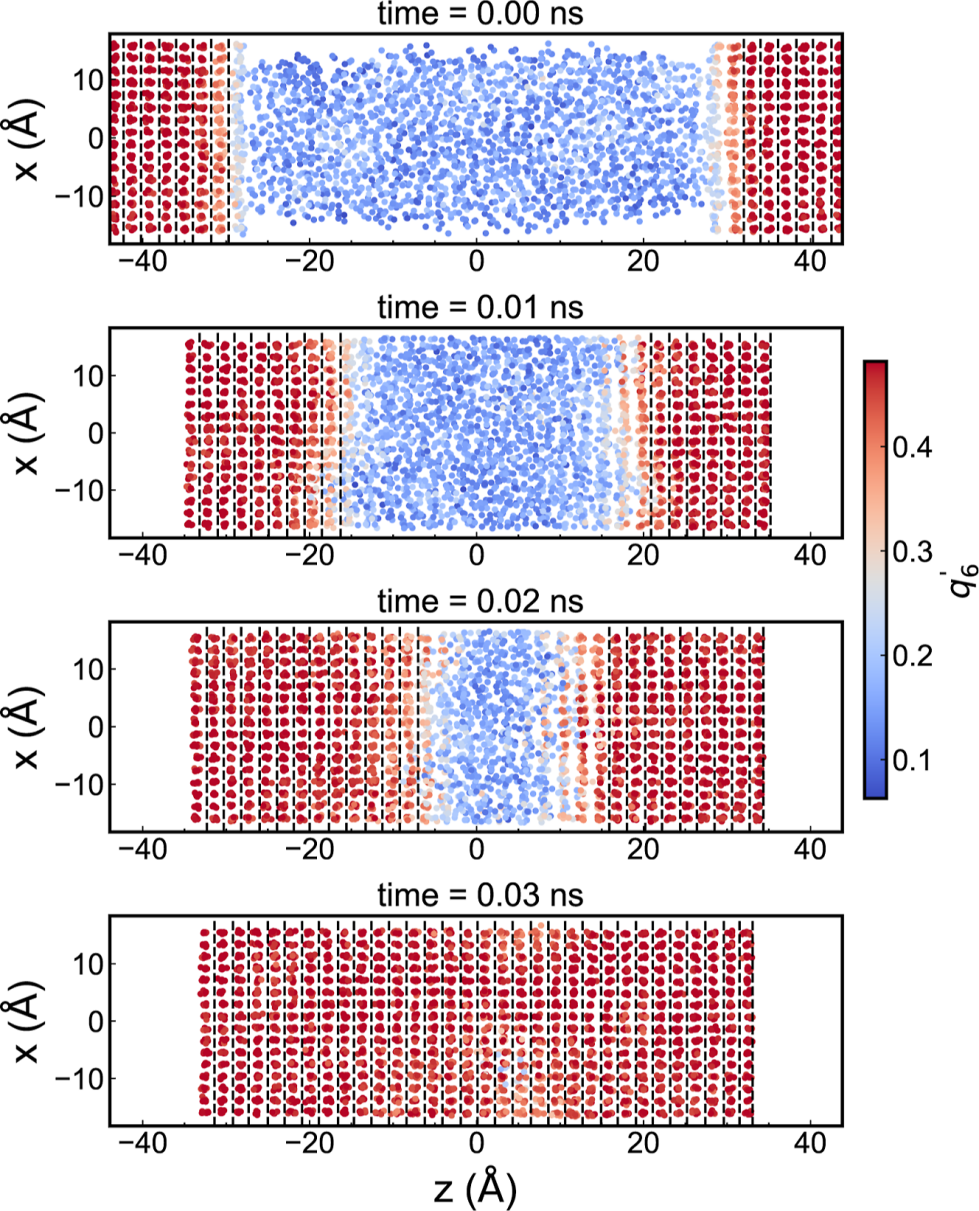
Scatter plot of the *q*_6_^′^ parameter for supercooled aluminum at a temperature of 800 K, pressure of 1 atm and different times. The temperature is 58 K below the standard melting point.^29,41^ Warmer colors (red) denote atoms in the solid state, while cooler colors (blue) indicate atoms in the liquid state. Vertical dashed lines show the central spacing between two crystallized layers.

The individual analysis of per-atom Gibbs free energy revealed distinct pathways when the energy is projected along the order parameter *q*_6_^′^ (see Figure S4). To classify the per-atom changes in Gibbs energies with respect to the reaction coordinate *q*_6_^′^, we employed the k-means clustering technique (see Figure 3). Using the unsupervised k-means method,^42^ we quantified the frequency of each pattern in the *g*/*q*_6_^′^ phase space. We set the number of clusters to four, which proved to be sufficient for distinguishing among the different types of Gibbs energy trajectories: barrierless, drop-then-barrier, barrier-only, and barrier-then-drop.

**Figure 3:**
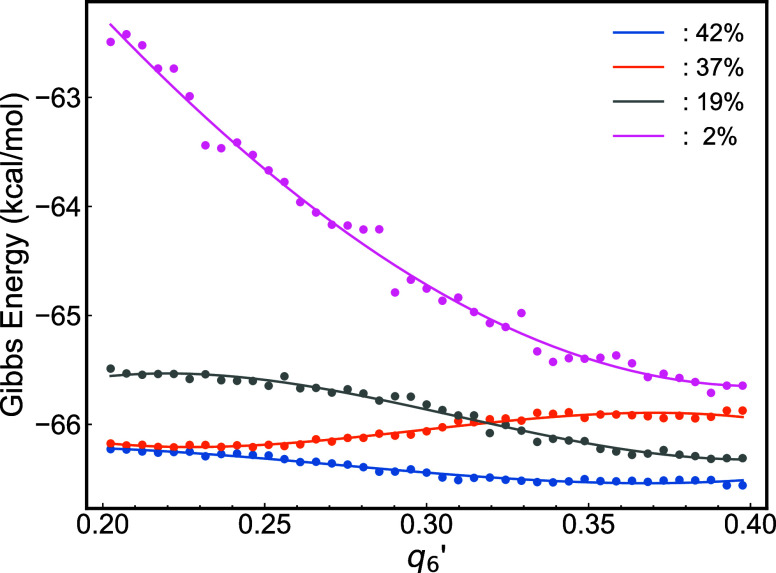
Per-atom Gibbs free energy changes along the *q*_6_^′^ reaction coordinate of supercooled aluminum at 800 K and 1 atm. The different data sets represent the four crystallization pathways identified through unsupervised k-means clustering. Typical pathways can be found in Supporting Information (Figure S3).

One of the free energy pathways exhibits the highest decrease in per-atom Gibbs free energy, approximately 2.5 kcal/mol. However, only 2% of the atoms contribute to this pathway. In contrast, the most common pathway is observed in 42% of the atoms and is characterized by a gradual decline in the per-atom Gibbs free energy. The other two pathways show a small decrease in free energy as they transition from the liquid to the solid phase (19%), and one features a small activation barrier of ∼0.5 kcal/mol (37%), equivalent to 0.4 *k*_B_ *T* at 800 K, making this barrier easily accessible at this high temperature.

Overall, for the crystallization direction (*q*_6_^′^ increasing), the patterns illustrated in Figure 3 predominantly show a consistent decrease in the per-atom Gibbs free energy. This supports a barrierless pathway for the crystallization of supercooled aluminum. The lack of a significant activation barrier in the forward direction of crystallization aligns with the ultrafast growth rate of aluminum, which can reach up to 67 m/s in our simulations. This value is consistent with previous findings by Sun et al.,^2^ who explored the origin of barrierless crystallization through computer simulations and energy minimization techniques. Here, we demonstrate the barrierless mechanism by explicitly analyzing the per-atom Gibbs free energies in conjunction with unsupervised clustering methods.

Figure 4 illustrates the average per-atom enthalpy (Δ*H*), entropy (*T*Δ*s*), and Gibbs free energy (Δ*G*), associated with the crystallization process along the [100] and [111] aluminum crystal planes. In both systems, there is a consistent decline in the enthalpic and entropic contributions as the atoms transition from the liquid (low *q*_6_^′^) to the solid phase (high *q*_6_^′^). Notably, there are no energy barriers as the atoms progress along the *q*_6_^′^ coordinates (from liquid to solid).

**Figure 4:**
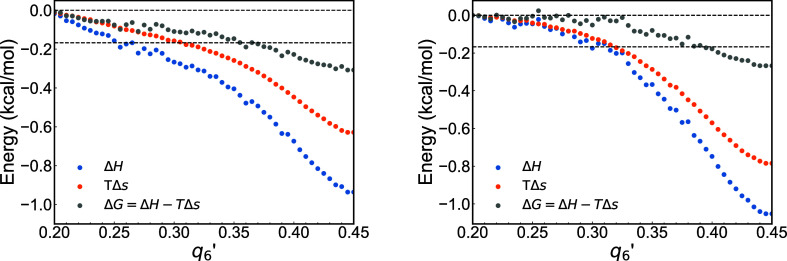
System enthalpy­(Δ*H*), entropy (*T*Δ*s*), and free energy (Δ*G*) along the [100]-left and [111]-right crystallographic directions. Dashed horizontal lines indicate the Gibbs free energy difference as defined by eq 4. The average values for the left panel correspond to the weighted average of the results reported in Figure 3.

The enthalpy decreases, indicating favorable energetic conditions for crystallization. However, this decrease is not linear with respect to *q*_6_^′^. There is a more pronounced drop in enthalpy for *q*_6_^′^ > 0.3, which corresponds to a region that is already highly ordered. This finding aligns with previous analyses of metals using embedded atom models.^2^

The entropy contribution displays a pattern similar to that of the enthalpy, and the entropy does not decrease linearly with *q*_6_^′^. We observe a significant decrease in entropy rate toward the end of the crystallization process. The Gibbs free energy, which is calculated as the difference between enthalpy and entropy, shows a similar downward trend, confirming that the process is energetically favorable. This observation further supports our conclusion that crystallization occurs without significant energy barriers. Furthermore, we do not find notable directional preference between the [100] and [111] crystallographic axes (cf. Figures 4-left and right). Figure 4 also shows that the free energy difference between the solid and liquid phases is consistent across the [100] and [111] systems, and within 0.1 kcal/mol of the free energy difference estimated using eq 4, confirming the validity of our per-atom analysis of the free energy and entropy.

To evaluate the robustness of our calculations, we conducted additional simulations of the silicon (Si) liquid–solid interfaces. These simulations were performed with the ReaxFF model developed in ref 43. According to that reference, the melting temperature of Si is reported to be 2423 ± 2 K. We performed our simulations at 1 atm and 2000 K, resulting in a moderate undercooling of 423 K.

Figure S9 in the Supporting Information illustrates that silicon exhibits rapid, barrierless crystallization, similar to aluminum. The speed of the [111] liquid–solid interface is approximately 30 m/s. Previous works derived relationship for the growth speed *v* = *a* exp­(−*b*/Δ*T*)­Δ*T* ^2/3^. Using the parameters reported in references Fabiyi et al.^44^ and Mishra et al.,^45^ this equation predicts a speed of ∼mm/s for undercoolings between 2 and 4 K. This prediction is consistent with experimental estimates of the speed for Czochralski growth, which typically yield rates of 10 μm/s at undercoolings of approximately 1.5 K (refer to Fabiyi et al.^44^ for further discussion).

We applied this equation to estimate the growth rate in our simulations, accounting for a substantial undercooling of 423 K. The resulting speed of about 18 m/s aligns with the order of magnitude computed in our simulations. This indicates that our simulations yield realistic results and that the large speeds, on the order of meters per second, are consistent with the barrierless crystallization growth (see Figure S9).

### The Crystal Growth of Alumina

In this section, we expand our analysis to alumina, which consists of two types of atoms: aluminum and oxygen. The cutoff for *r*_m_ in the *s*_2_ calculation is the average of 1.4 times^15^ the lattice constant in the basal plane^27^ (*r*_m_ = 1.4 × 4.76 = 6.66 Å). We use the partial RDFs, *g*_*ii*_ to calculate the *s*_2_, namely we chose *i* = aluminum or *i* = oxygen atoms individually to evaluate their local ordering in relation to atoms of the same type since the partial RDF. We fitted *f*_0_ to account for the missing contributions to the entropy, using eq 5. All the calculations reported below correspond to 2000 K and *f*_0_ = 1.50.

Previous simulations of silica^31^ successfully observed crystallization at 40 GPa while overcoming glassification. In our previous work on alumina,^29^ we demonstrated that crystallization can occur on the nanosecond time scale under high-pressure conditions, ∼10 GPa. This pressure allows for the completion of the crystallization process within 1 ns through direct coexistence simulations,^29,39^ enabling the examination of individual atomic trajectories leading to crystallization events.

Figure 5 illustrates the Gibbs free energy of aluminum and oxygen atoms in supercooled alumina during the crystallization process. Figure S5 shows simulation snapshots of the crystal growth process. This analysis is conducted along the *q*_6_^′^ coordinate at a temperature of 2000 K and a pressure of 10 GPa. This temperature is below the melting point of alumina modeled with ReaxFF, which is 2630 K at 10 GPa.^29^ To ensure the accurate convergence of values at both the initial and final points of crystallization, the range of the order parameter *q*_6_^′^ is set between 0.13 and 0.42 (see Figure S6 for the histograms of the *q*_6_^′^ order parameter and Figures S7 and S8 for examples of free energy pathways projected along the order parameter). This interval corresponds to complete crystallization and differs slightly from that of metallic aluminum due to variations in lattice structure.

**Figure 5:**
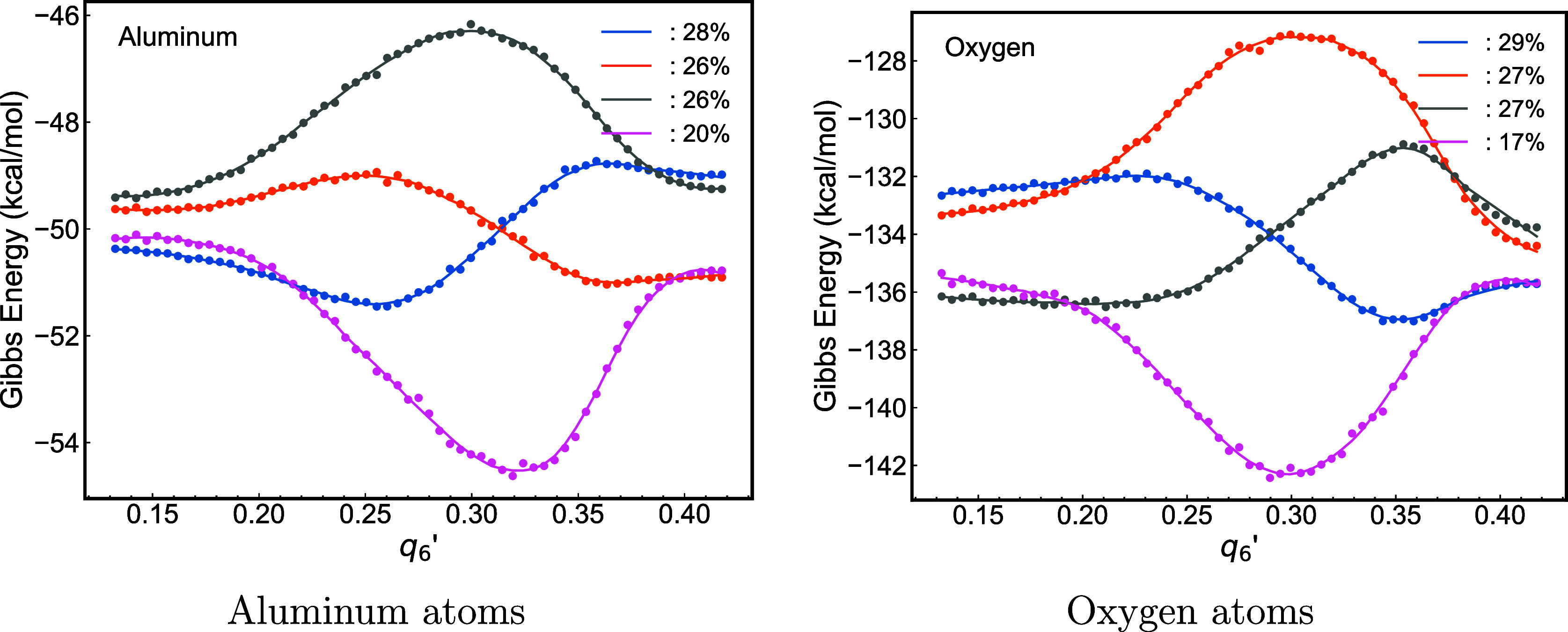
Per-atom Gibbs energy change along the “reaction” coordinate *q*_6_^′^ of supercooled alumina at 2000 K and 10 GPa. The left and right panels show the results for aluminum and oxygen atoms, respectively. Typical pathways for each of the four pathways considered in this analysis are reported in Figures S6 and S7.

In contrast to the barrierless crystal growth process in metallic aluminum, the oxygen and aluminum energy trajectories in alumina feature energy barriers. The Gibbs energy of aluminum atoms indicates a forward barrier of approximately 3 kcal/mol (represented by the blue and gray lines in Figure 5-left), while oxygen atoms exhibit a higher barrier of around 6 kcal/mol (shown by the orange and gray lines in Figure 5-right). These energies are of the order of the thermal energy at 2000 K, explaining the possibility of observing the crystallization process at this high temperature. The starting point of crystallization, corresponding to the low order parameters (*q*_6_^′^ = 0.13), reveals two initial free energy values. For aluminum atoms, the differences in these values are relatively small, about 1 kcal/mol. For oxygen atoms, the differences are more substantial, exceeding 3 kcal/mol, but overall these energies are below the thermal energy at 2000 K, and hence, they are not significant.

The presence of energy barriers slows down the rate of crystallization. For alumina at 2000 K and 10 GPa, our analysis indicates that the speed of crystal growth is approximately 13 m/s. This rate is notably slower than metallic aluminum, despite alumina having a higher diffusion rate at this elevated temperature. Crystal growth at high pressure occurs significantly faster than under standard conditions, primarily due to the elevated melting temperature, which enables direct observation of crystallization events on subnanosecond time scales. Investigating systems at lower pressures offers significant potential for advancing the understanding of alumina crystallization. However, simulating these low-pressure conditions poses computational challenges that must be addressed to ensure reliable results. Therefore, the results presented here provide insights into the microscopic mechanisms of crystal growth and the reasons behind the slow crystal growth rate observed in experiments and simulations of alumina.

Figure 6 illustrates the mean per-atom enthalpic and entropic contributions to the Gibbs free energy for the two alumina atoms types: aluminum and oxygen. The enthalpy exhibits a smooth decrease for the aluminum atoms without significant energy barriers, suggesting that these atoms do not encounter significant energetic hindrances during the crystallization process. This indicates that aluminum atoms undergo a barrierless transition, with their enthalpic energy pathway largely decreasing. Consistent with the behavior observed in the crystal growth of aluminum, we find an increase in the rate of enthalpy reduction for *q*_6_^′^ > 0.3. The variations in enthalpy are balanced by changes in the entropic contribution, which varies at a similar rate. As a result, the Gibbs free energy remains nearly constant along the “reaction” coordinate.

**Figure 6:**
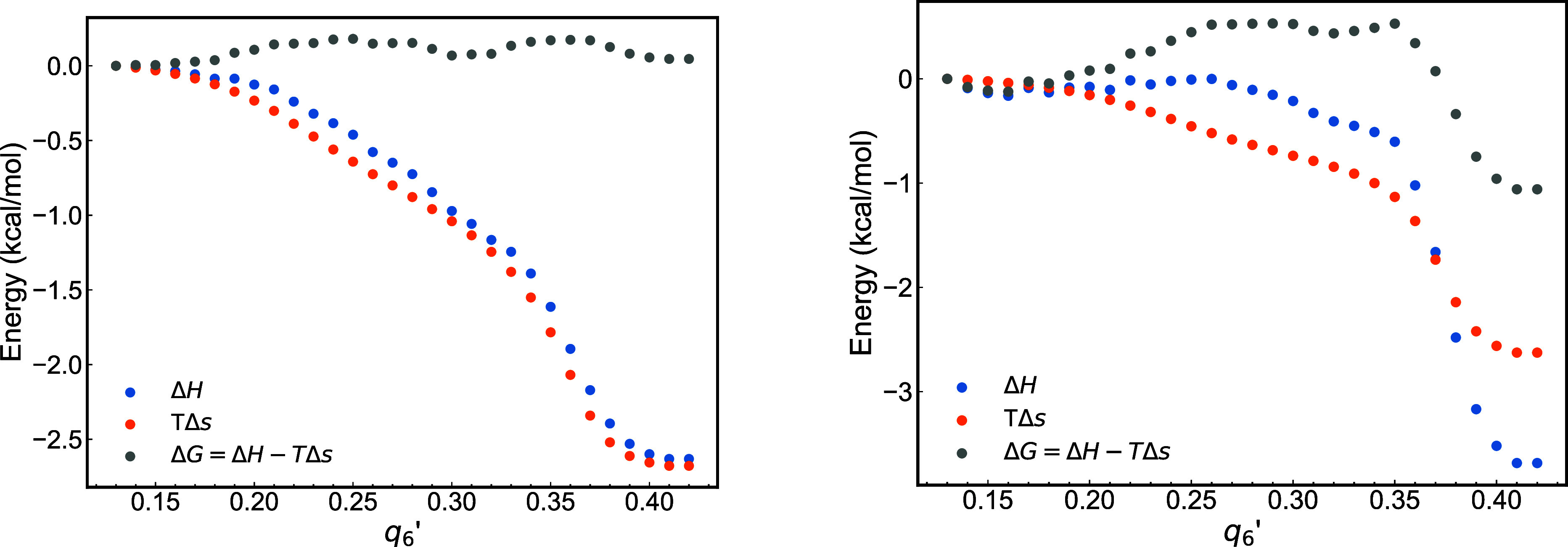
Average enthalpic and entropic contributions to the Gibbs free energy of supercooled alumina during the crystallization process. (Left panel) aluminum. (Right panel) oxygen. The average values were obtained from a weighted average of the results reported in Figure 5.

In contrast to the scenario for aluminum atoms, the free energy analysis of the oxygen atoms (see Figure 6 on the right) reveals the presence of an energy barrier, approximately 1 kcal/mol. This suggests that oxygen atoms require activation to initiate crystallization. The differences in the energy pathways for aluminum and oxygen imply that while aluminum atoms move more freely and readily toward crystallization, oxygen atoms encounter greater resistance. This resistance may slow their incorporation into the crystal structure, potentially representing a rate-determining step in the crystallization process. Additionally, the enthalpic contribution shows a significant decrease at *q*_6_^′^ > 0.35, indicating that the main energy transfer process occurs when the oxygen atoms are nearly fully incorporated into the evolving crystal structure.

To gain a microscopic understanding of the interfacial crystal growth of alumina, we analyzed the simulation trajectories to investigate how alumina and oxygen atoms are sequentially incorporated into the crystal phase. We focused on the (0001) face of alumina as the growth direction for the interface. We indexed each crystal layer based on their interlayer spacing along the *z*-direction (see Figure 8). The layers are labeled as 0, 1, 2, 3... for aluminum (and −1, 0, 1, 2, 3... for oxygen), with the base reference layer 0 corresponding to *z̅* = 0. These indices are defined with bold numbers in Figure 8.

In Figure 7, we present the per-layer averaged *q*_6_^′^ during the crystallization of alumina, focusing on the aluminum layer with an average z-coordinate of 9.85 Å, which is the fourth layer overall (see Figure 8). The analysis of the time dependence of the oxygen layers (indicated by dashed lines) reveals insights into the crystallization mechanism. The second layer of oxygen is incorporated after the third layer of oxygen, and the latter after the fourth layer of oxygen, showing that a higher *z̅*-coordinate values correspond to faster crystallization. This observation is connected to the fact that the fourth layer is closer to the initial liquid/solid interface. Aluminum incorporation into the crystal occurs only after the fourth layer of oxygen has been fully integrated. This result suggests a sequential crystallization process. Specifically, the aluminum layer begins to crystallize after the nearest front oxygen layer but before its nearest oxygen layer. This sequence is consistently observed throughout the entire crystallization process. The quicker localization of the oxygen atoms into their equilibrium positions is consistent with their higher diffusion rates. Rymzhanov et al.^46^ reported similar findings when simulating the recrystallization of surface damage in MgO and Al_2_O_3_. In Al_2_O_3_, it was found that oxygen atoms are incorporated into the crystal structure faster than the metallic atoms.

**Figure 7:**
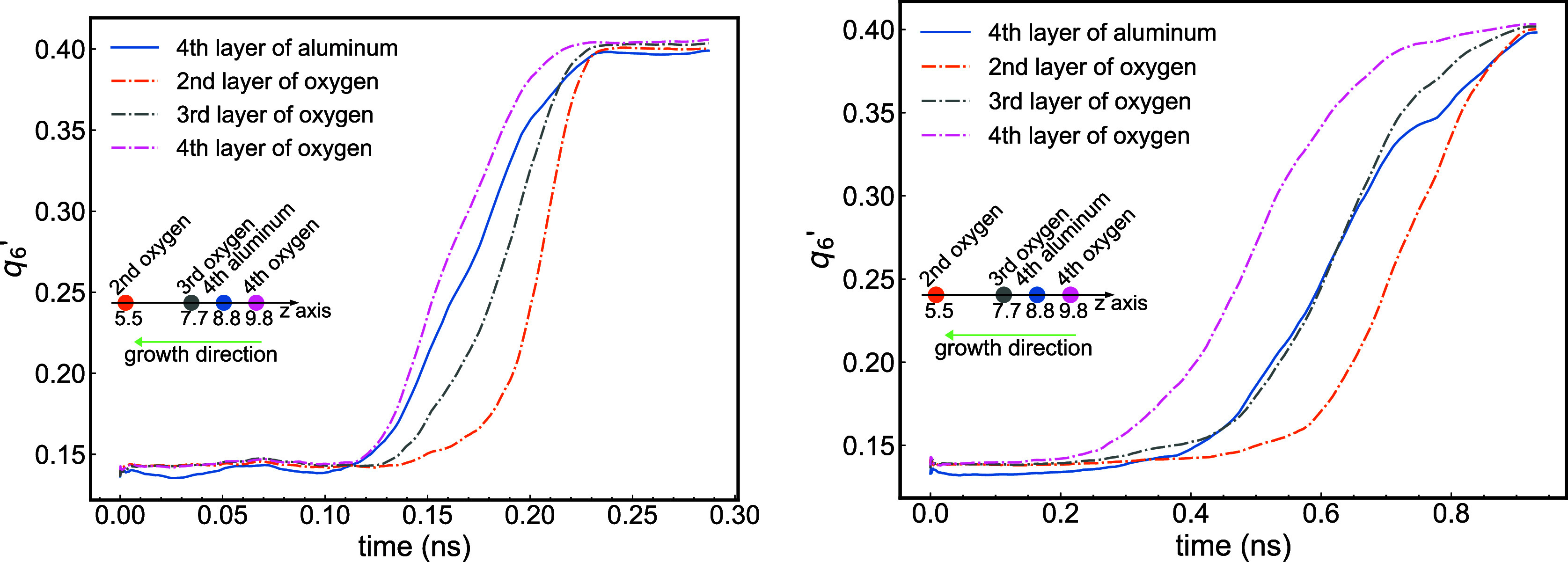
Per-layer average of *q*_6_^′^ as a function of time for supercooled alumina at 2000 K and (left panel) 10 and (right panel) 5 GPa. The solid line represents the aluminum layer, while the dashed lines represent the oxygen layers. Layer numbers start from left to right based on the snapshot shown in Figure 8 and time *t* = 0.3 ns.

**Figure 8:**
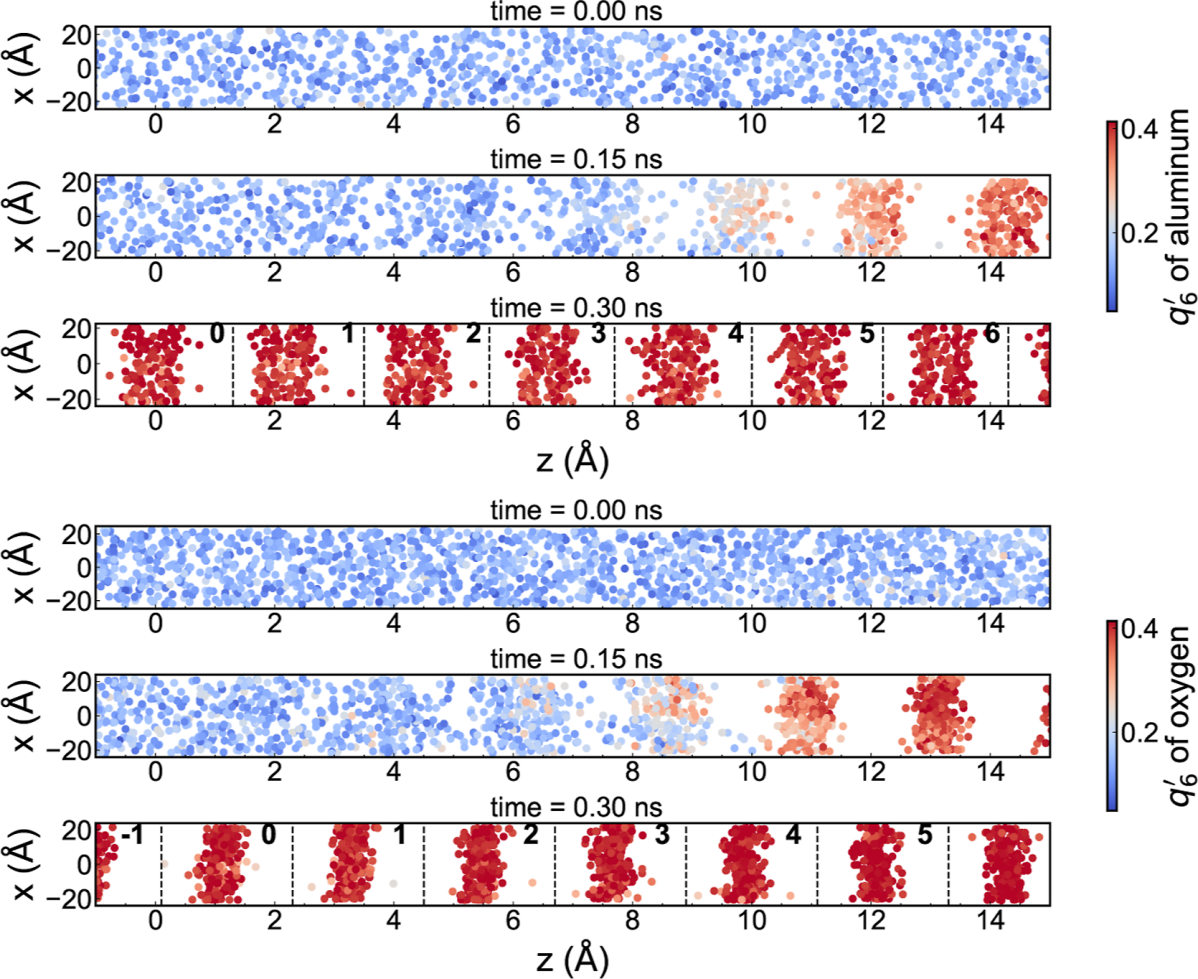
Snapshots of the simulations along the *x*–*z* plane, color-coded according to the *q*_6_^′^ parameter. The upper and lower panels represent the aluminum and oxygen atoms, respectively. The results correspond to a pressure of 10 GPa.

We performed additional computations at other pressures. Figure 9 shows the free energy curves projected on the oxygen atoms at 5, 10, and 20 GPa. The free energy changes significantly with the pressure developing an energy barrier at lower pressures. The barrier reduces the speed of the crystallization front, 10.71, 7.06, and 2.33 m/s for 20, 10, and 5 GPa, respectively. However, the sequential mechanism of incorporating oxygen and aluminum remains the same (see Figure 7).

**Figure 9:**
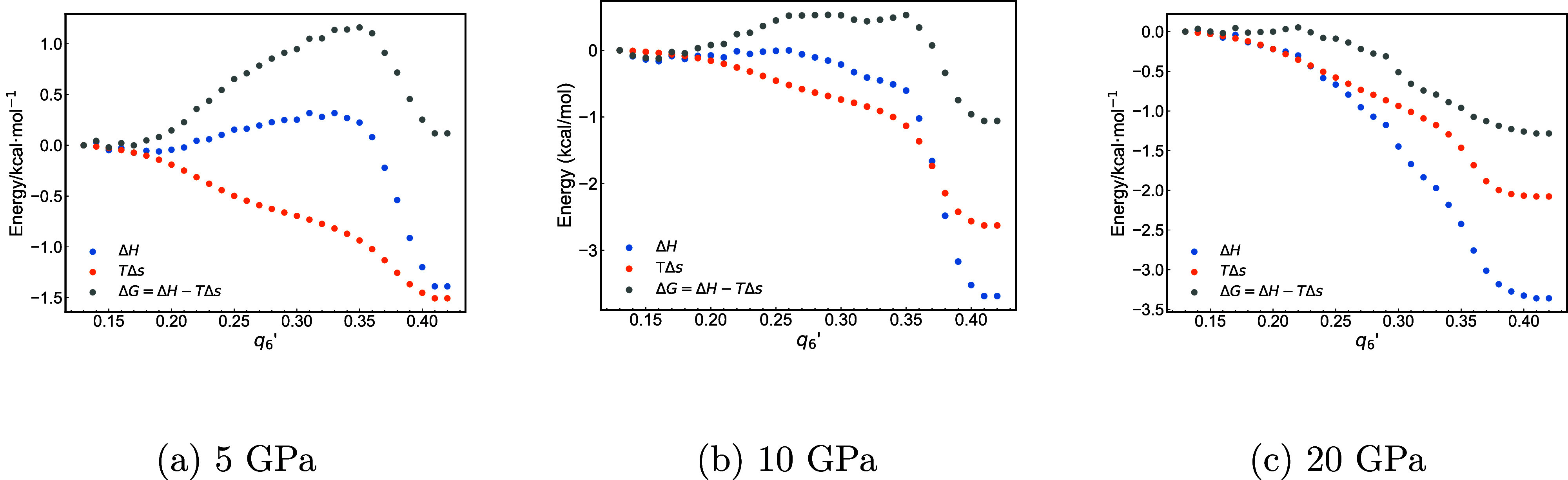
System enthalpy (Δ*H*), entropy (*T*Δ*S*), and free energy (Δ*G*) along the oxygen atoms of alumina crystallographic direction at 2000 K and at 5, 10, and 20 GPa, respectively.

A significant difference between the alumina and aluminum systems is the variation in atomic charge observed in the alumina crystallization. As crystallization proceeds, the absolute charges of aluminum and oxygen atoms increase. These changes in atomic charge are correlated with the ongoing crystallization process. Figure 10 shows a scatter plot with charge on the *x*-axis and Gibbs free energy on the *y*-axis for the atoms that underwent crystallization. The Gibbs free energy of the oxygen atoms is significantly influenced and correlated with changes in the atomic charge. In contrast, the Gibbs free energy of the aluminum atoms features a weak correlation with the atomic charge (see left panel of Figure 10), reflected in a small gradient and an R-squared value close to zero.

**Figure 10:**
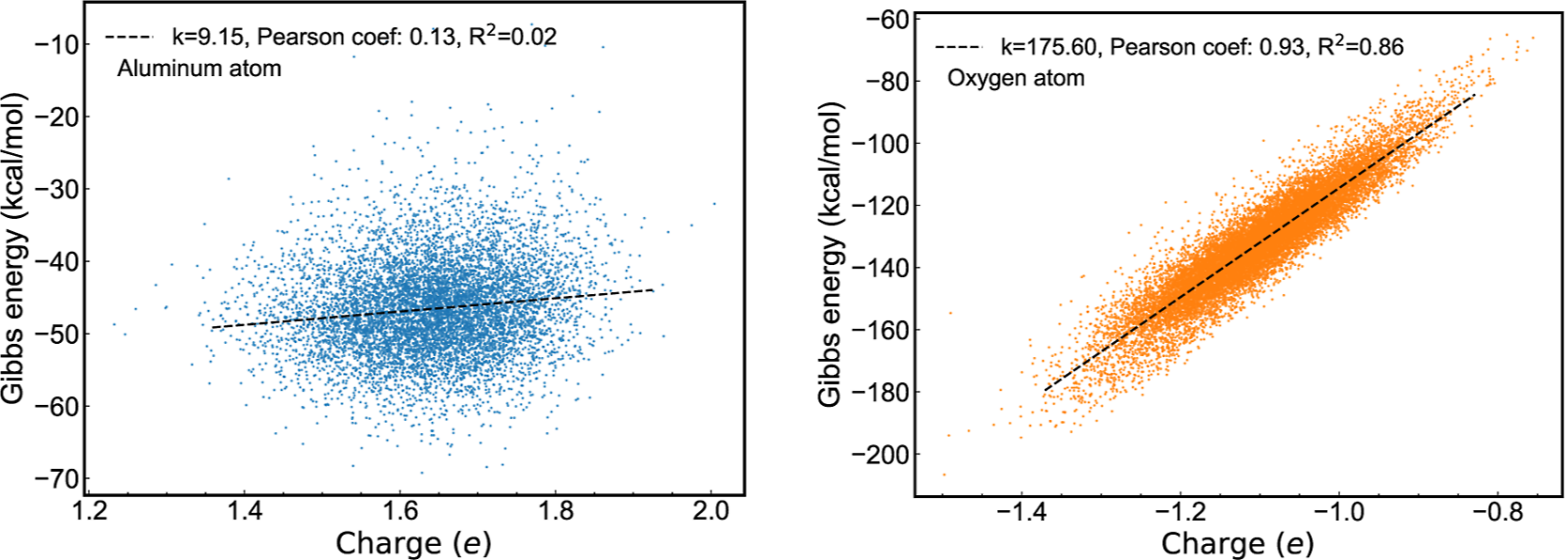
Scatter plots illustrating the correlation between atomic charge and Gibbs free energy of aluminum (left) and oxygen (right) atoms in a supercooled liquid-crystal alumina system at 2000 K and 10 GPa.

In contrast, the oxygen atoms (see right panel of Figure 10) show a strong correlation in gradient and R-squared values, indicating significant correlations between the Gibbs free energy and atomic charge during crystallization. Notably, aluminum has a positive Pearson correlation coefficient, indicating that its Gibbs energy increases with increasing charge, with a larger positive charge corresponding to the solid phase. This results in a higher Gibbs energy after crystallization. On the other hand, oxygen exhibits the opposite trend. A more negative charge (observed in the solid phase) leads to an overall decrease in the oxygen Gibbs energy. The strong dependence of Gibbs energy on the crystallization direction of oxygen atoms may explain the crystallization sequence, where aluminum crystallizes only after one of its neighboring oxygen atoms has ordered, as oxygen has a lower Gibbs energy. We want to emphasize that the changes in atomic charge described here can be modeled using the ReaxFF force field, which has been parametrized with Density Functional Theory (DFT) computations. While the original ReaxFF parametrization did not include explicit training of atomic charges based on DFT data, later versions such as eReaxFF^47^ have incorporated explicit charge training using DFT results. This shift in the magnitude of the atomic charge effect is not accounted for in other commonly used models for simulating metals, such as Embedded Atom Models.

We also examined the case of silica (SiO_2_) using the force field described in ref 43. However, we did not observe any crystal growth at pressures of 40 GPa and at 2000 K. This lack of growth may be due to limitations imposed by the cooling rate for this system, as well as the inherently slow crystallization kinetics of silica compared to alumina and the specific force field used. Therefore, further investigation of silica is warranted.

## Conclusions

We have investigated the crystallization pathways of supercooled aluminum and alumina from their melts using reactive force fields and direct liquid–solid coexistence molecular dynamics simulations. Our approach involves analyzing thousands of atomic trajectories, which we classify and sort into different free energy pathways using a weighted order parameter *q*_6_^′^, combined with an unsupervised clustering algorithm. Additionally, we have introduced a methodology for computing the per-atom Gibbs free energy along the reaction coordinate *q*_6_^′^. This method relies on quantifying the two-body excess entropy and local atomic volumes using Voronoi tessellation techniques.

We have identified three major pathways for the crystallization of solid aluminum. The weighted average of these free energy pathways suggests that the process occurs without a significant energy barrier. This finding is consistent with the ultrafast crystallization and high growth rates observed in metals, approximately 10^2^ m/s. Our analysis of enthalpic and entropic contributions indicates that both contributions exhibit a nonlinear dependence with the *q*_6_^′^ “reaction” coordinate, featuring a significant drop during the final stages of the crystallization process when the atoms are incorporated into the crystal structure.

Using the free energy approach introduced in this study, we investigated the crystallization mechanism of alumina. We selected a high-pressure thermodynamic state (10 GPa), which allows us to observe crystal growth in simulations at nanosecond time scales. Despite the stronger undercooling, the crystal growth rate of alumina is considerably slower, ∼13 m/s for (*T* – *T*_m_)/*T*_m_ = −0.24 vs 67 m/s for aluminum at (*T* – *T*_m_)/*T*_m_ = −0.075. The crystallization pathways of alumina, based on analyses of thousands of atomic trajectories, feature energy barriers and a complex atomic sequence for the incorporation of atoms into the alumina crystal.

During the crystallization of alumina, one layer of oxygen atoms deposits on the crystal face more rapidly than aluminum atoms due to higher diffusion rates. A strong correlation between atomic charge and Gibbs energy is observed for oxygen, highlighting the importance of Coulombic interactions. The sequential crystallization process reported here illustrates that aluminum layers crystallize after adjacent oxygen layers are incorporated into the crystal via an activated process. The preferential ordering or site occupation of oxygen atoms within the alumina lattice provides a determining rate step, which we suggest is responsible for the low growth rates in these metal oxide interfaces.

We have demonstrated an increase in the activation free energy of alumina as the pressure decreases. The microscopic mechanisms we have identified remain qualitatively relevant even at lower pressures, specifically down to 5 GPa. It is anticipated that further reductions in pressure will lead to correspondingly slower interface growth rates. Slower rates align with experimental findings at standard pressure. The GPa pressure conditions explored in our study are relevant in geophysics. Extrapolating our results to standard pressure is not straightforward because the melting point decreases with decreasing pressure, and the interface speed may not change linearly with pressure. However, our work establishes a microscopic mechanism operating in the accessible high-pressure regime and provides an atomistic foundation for understanding alumina crystallization across broader pressure–temperature conditions. Future experimental validation at intermediate pressures could bridge our high-pressure findings with ambient conditions.

The combination of reactive (ReaxFF) simulations and free energy calculations offers a promising approach for studying the reactivity of complex aluminum-based materials and, more broadly, metal oxides. These methods may also be extended to explore other thermodynamic conditions, such as the crystal growth of metal oxide interfaces at lower pressures. Additionally, these studies can support mechanistic studies and complement efforts to develop materials relevant to energy applications.

## Supplementary Material



## Notes

Contains additional data on the potential energy of alumina during the crystallization process, Gibbs energy pathways identified through unsupervised clustering algorithms, histograms of the *q*_6_^′^ order parameter, and snapshots that illustrate the crystallization process of alumina (PDF)

## References

[1] Coriell S., Turnbull D. (1982). Relative roles of heat transport and interface rearrangement
rates in the rapid growth of crystals in undercooled melts. Acta Metall..

[2] Sun G., Xu J., Harrowell P. (2018). The mechanism
of the ultrafast crystal growth of pure
metals from their melts. Nat. Mater..

[3] Orava J., Greer A. L. (2014). Fast and slow crystal
growth kinetics in glass-forming
melts. J. Chem. Phys..

[4] Jia J., Liu D., Gao C., Ji G., Guo T. (2018). Preparation and mechanical
properties of short carbon fibers reinforced α-Al2O3-based composites. Ceram. Int..

[5] Bozorgan N., Shafahi M. (2015). Performance evaluation
of nanofluids in solar energy:
a review of the recent literature. Micro Nano
Syst. Lett..

[6] Rao J. K., Madhusudhan R., Rao T. B. (2022). Recent progress in stir cast aluminum
matrix hybrid composites: overview on processing, mechanical and tribological
characteristics, and strengthening mechanisms. J. Bio- Tribo-Corros..

[7] Chandel R., Sharma N., Bansal S. A. (2021). A review
on recent developments of
aluminum-based hybrid composites for automotive applications. Emergent Mater..

[8] Van
Duin A. C., Dasgupta S., Lorant F., Goddard W. A. (2001). ReaxFF:
a reactive force field for hydrocarbons. J.
Phys. Chem. A.

[9] Chenoweth K., Van Duin A. C., Goddard W. A. (2008). ReaxFF
reactive force field for molecular
dynamics simulations of hydrocarbon oxidation. J. Phys. Chem. A.

[10] Zhang Y. R., van Duin A. C., Luo K. H. (2018). Investigation
of ethanol oxidation
over aluminum nanoparticle using ReaxFF molecular dynamics simulation. Fuel.

[11] Li G., Niu L., Hao W., Liu Y., Zhang C. (2020). Atomistic insight into
the microexplosion-accelerated oxidation process of molten aluminum
nanoparticles. Combust. Flame.

[12] Hong S., Van Duin A. C. (2016). Atomistic-scale
analysis of carbon coating and its
effect on the oxidation of aluminum nanoparticles by ReaxFF-molecular
dynamics simulations. J. Phys. Chem. C.

[13] Joshi K. L., Psofogiannakis G., Van Duin A. C., Raman S. (2014). Reactive molecular
simulations of protonation of water clusters and depletion of acidity
in H-ZSM-5 zeolite. Phys. Chem. Chem. Phys..

[14] Dumortier L., Chizallet C., Creton B., de Bruin T., Verstraelen T. (2024). Managing Expectations
and Imbalanced Training Data in Reactive Force Field Development:
An Application to Water Adsorption on Alumina. J. Chem. Theory Comput..

[15] Piaggi P. M., Parrinello M. (2017). Entropy based fingerprint for local crystalline order. J. Chem. Phys..

[16] Lin J.-F., Degtyareva O., Prewitt C. T., Dera P., Sata N., Gregoryanz E., Mao H.-k., Hemley R. J. (2004). Crystal structure
of a high-pressure/high-temperature phase of alumina by in situ X-ray
diffraction. Nat. Mater..

[17] Santanach J. G., Weibel A., Estournès C., Yang Q., Laurent C., Peigney A. (2011). Spark plasma sintering of alumina: Study of parameters,
formal sintering analysis and hypotheses on the mechanism(s) involved
in densification and grain growth. Acta Mater..

[18] Anderson O. L., Isaak D., Oda H. (1992). High-temperature
elastic constant
data on minerals relevant to geophysics. Rev.
Geophys..

[19] Shen G., Lazor P. (1995). Measurement of melting
temperatures of some minerals under lower
mantle pressures. J. Geophys. Res.: Solid Earth.

[20] Stixrude L., Lithgow-Bertelloni C. (2011). Thermodynamics of mantle minerals - II. Phase equilibria. Geophys. J. Int..

[21] Chenoweth K., Van Duin A. C., Persson P., Cheng M.-J., Oxgaard J., Goddard W. A. (2008). Development and application of a
ReaxFF reactive force field for oxidative dehydrogenation on vanadium
oxide catalysts. J. Phys. Chem. C.

[22] Hong S., van Duin A. C. (2015). Molecular dynamics simulations of the oxidation of
aluminum nanoparticles using the ReaxFF reactive force field. J. Phys. Chem. C.

[23] van
Duin A. C., Baas J. M., Van De Graaf B. (1994). Delft molecular
mechanics: a new approach to hydrocarbon force fields. Inclusion of
a geometry-dependent charge calculation. J.
Chem. Soc., Faraday Trans..

[24] Shchygol G., Yakovlev A., Trnka T., Van Duin A. C., Verstraelen T. (2019). ReaxFF parameter
optimization with Monte-Carlo and evolutionary algorithms: guidelines
and insights. J. Chem. Theory Comput..

[25] Zhang Q., Çaǧın T., Van Duin A., Goddard W. A., Qi Y., Hector L. G. (2004). Adhesion and nonwetting-wetting
transition in the Al/α-Al2O3
interface. Phys. Rev. B.

[26] Zemann J. (1965). Crystal structures,
Vol. 1 by RWG Wyckoff. Acta Crystallogr..

[27] Lewis J., Schwarzenbach D., Flack H. (1982). Electric field gradients
and charge
density in corundum, α-Al2O3. Acta Crystallogr.
A.

[28] Ong S. P., Richards W. D., Jain A., Hautier G., Kocher M., Cholia S., Gunter D., Chevrier V. L., Persson K. A., Ceder G. (2013). Python Materials Genomics
(pymatgen): A robust, open-source python
library for materials analysis. Comput. Mater.
Sci..

[29] Zhao H., Bresme F. (2024). Melting Point and Crystal
Growth Kinetics of Metals
and Metal Oxides Using Reactive Force Fields: The Case of Aluminum
and Alumina. J. Chem. Theory Comput..

[30] Mortier W. J., Ghosh S. K., Shankar S. (1986). Electronegativity-equalization
method
for the calculation of atomic charges in molecules. J. Am. Chem. Soc..

[31] Niu H., Piaggi P. M., Invernizzi M., Parrinello M. (2018). Molecular
dynamics simulations of liquid silica crystallization. Proc. Natl. Acad. Sci. U.S.A..

[32] Steinhardt P. J., Nelson D. R., Ronchetti M. (1983). Bond-orientational order in liquids
and glasses. Phys. Rev. B.

[33] Mickel W., Kapfer S. C., Schröder-Turk G. E., Mecke K. (2013). Shortcomings
of the bond orientational order parameters for the analysis of disordered
particulate matter. J. Chem. Phys..

[34] Lindemann F. (1910). The calculation
of molecular vibration frequencies Phys. Z.

[35] Rycroft C. H. (2009). VORO++:
A three-dimensional Voronoi cell library in C++. Chaos.

[36] Baranyai A., Evans D. J. (1989). Direct entropy calculation from computer simulation
of liquids. Phys. Rev. A.

[37] Wallace D. C. (1987). On the
role of density fluctuations in the entropy of a fluid. J. Chem. Phys..

[38] Laird B. B., Haymet A. D. J. (1992). Calculation of
the entropy from multiparticle correlation
functions. Phys. Rev. A.

[39] Huang Y., Widom M. (2024). Entropy approximations for simple
fluids. Phys.
Rev. E.

[40] Broughton J., Gilmer G., Jackson K. A. (1982). Crystallization
rates of a Lennard-Jones
liquid. Phys. Rev. Lett..

[41] Yan R., Sun W., Ma S., Jing T., Dong H. (2020). The orientation dependence
of liquid ordering at α-Al2O3/Al solid–liquid interfaces:
A molecular dynamics study. Comput. Mater. Sci..

[42] Jain, A. K. ; Dubes, R. C. Algorithms for Clustering Data; Prentice-Hall, Inc.: USA, 1988.

[43] Nayir N., Van Duin A. C. T., Erkoc S. (2019). Development of the
ReaxFF Reactive
Force Field for Inherent Point Defects in the Si/Silica System. J. Phys. Chem. A.

[44] Fabiyi V. A., Richmond T., Helenbrook B. T., Paek E. (2022). Molecular dynamics
determination of Two-dimensional nucleation kinetic coefficient for
modeling the faceted growth of Si (1 1 1) from an undercooled melt. J. Cryst. Growth.

[45] Mishra S. S., Chuang L.-C., Nozawa J., Maeda K., Morito H., Fujiwara K., Duffar T. (2024). Vicinal (111)
surfaces at Si solid-liquid
interface during unidirectional solidification. Scr. Mater..

[46] Rymzhanov R., Medvedev N., O’Connell J. H., Janse van Vuuren A., Skuratov V., Volkov A. (2019). Recrystallization as
the governing
mechanism of ion track formation. Sci. Rep..

[47] Islam M. M., Kolesov G., Verstraelen T., Kaxiras E., Van Duin A. C. T. (2016). eReaxFF:
A Pseudoclassical Treatment of Explicit Electrons within Reactive
Force Field Simulations. J. Chem. Theory Comput..

