# Supplementary material for: Recrystallization Mechanisms of Aluminum and Aluminum Oxide Interfaces through Reactive Simulations
Source: JACS Au. 2025 Sep 10;5(9):4625–35. doi: 10.1021/jacsau.5c01074 (PMC12458036; doi:10.1021/jacsau.5c01074)
Supplement: Supplementary file 1 [file au5c01074_si_001.pdf]

Supporting Information: Recrystallization Mechanisms of Aluminum and Aluminum Oxide Interfaces Through Reactive Simulations

Hao Zhao[†,‡] and Fernando Bresme[*,†]

†*Department of Chemistry, Molecular Sciences Research Hub, Imperial College, London, W12 0BZ, United Kingdom*

‡*State Key Laboratory of Multiphase Flow in Power Engineering, Xi'an Jiaotong University, Xi'an, Shaanxi, 710049, China*

E-mail: f.bresme@imperial.ac.uk

Simulation details

The preparation of our single-phase simulation involved three steps: first, a 5000-step NPT simulation was conducted to heat or cool the system by 10 K. This was followed by a 5000-step NPT equilibrium run. It was found that this heating/cooling rate (3 K/ps) balances simulation efficiency with stability. The additional 5000-step NPT run allows the system to equilibrate. Finally, a 10,000-step NVT simulation was performed for sampling, with bulk phase configuration data saved every 1,000 steps. The trajectories included atomic coordinates, velocities, and charges, which were directly used to analyze the solid/liquid interface system. Uncertainty estimates were obtained from 10 independent simulations, each initiated from a saved configuration with a different initial speed.

The Nosé-Hoover method[S1,S2] was used for both thermostat and barostat with damping constants of 50 fs for the temperature and 250 fs for the pressure. The heating/cooling rate was 3 K/ps, slow enough to prevent excessive heating when simulating the ReaxFF force field.[S3] More details about the simulation setup can be found in our previous work.[S4]

The NPT ensemble simulations were performed using a temperature damping time of 200 time steps (50 fs), and a pressure damping time of 1000 time steps (250 fs). A time step of 0.25 fs was used for all the MD simulations. This time step is a good balance between simulation efficiency and accuracy to integrate the fast degrees of freedom associated with the dynamic bond order of ReaxFF. All MD simulations were conducted using the LAMMPS molecular dynamics simulator[S5] (version 2 Aug 2023) with the ReaxFF package[S6] and visualized with the OVITO Basic.

The schematic below provides a summary of the computational approach used in this work.

Additional Data

1. Potential energy changes of aluminum during the crystallization process.

2. q_6' histograms of aluminum and alumina crystallization.

3. Gibbs free energy pathways identified through unsupervised k-means clustering.

4. The q_6' snapshots of alumina crystallization at different times.

References

(S1) Nosé, S. A molecular dynamics method for simulations in the canonical ensemble. *Molecular physics* **1984**, *52*, 255–268.

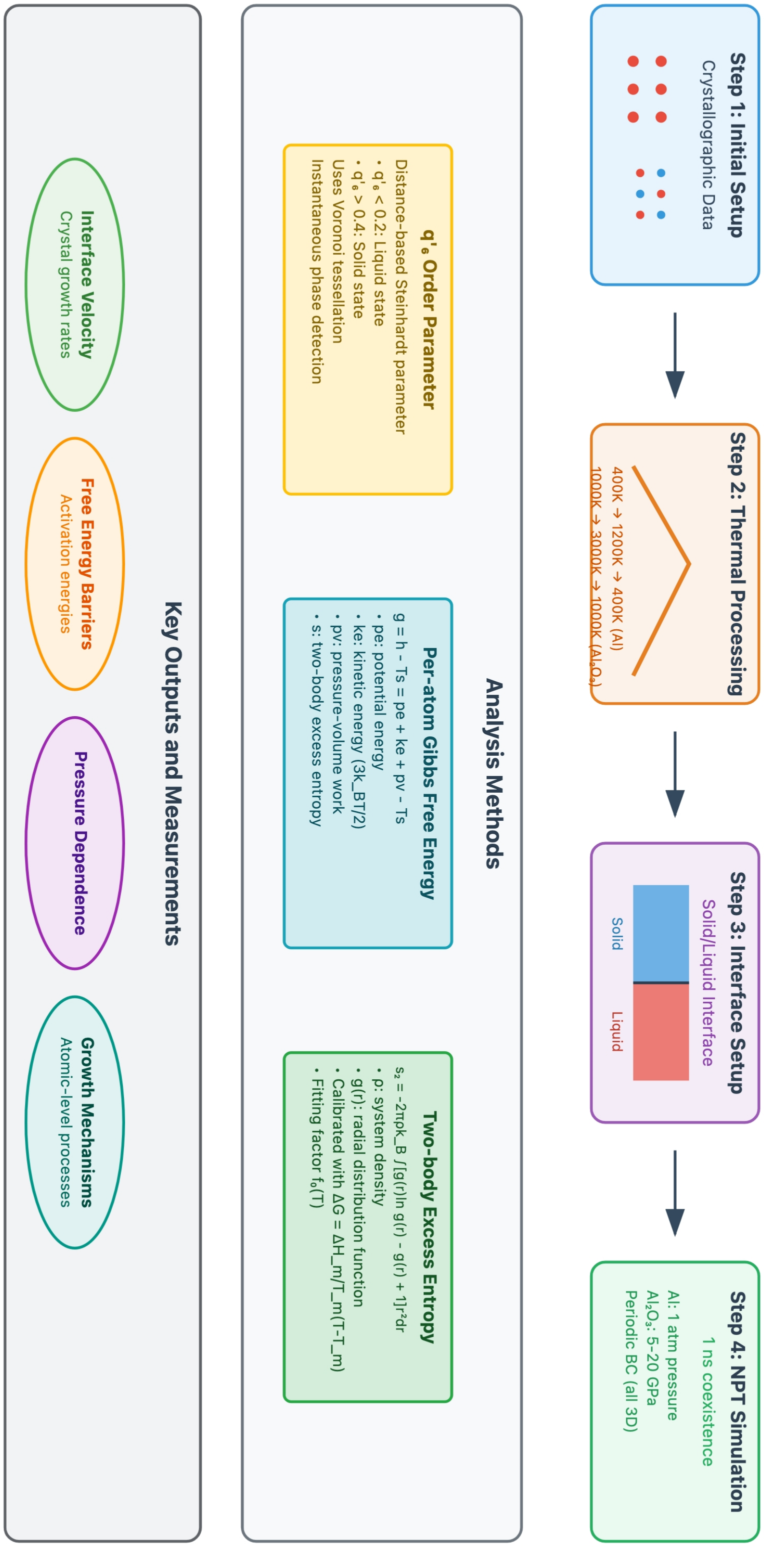

Figure S1: Workflow diagram illustrating the simulation method employed in this work.

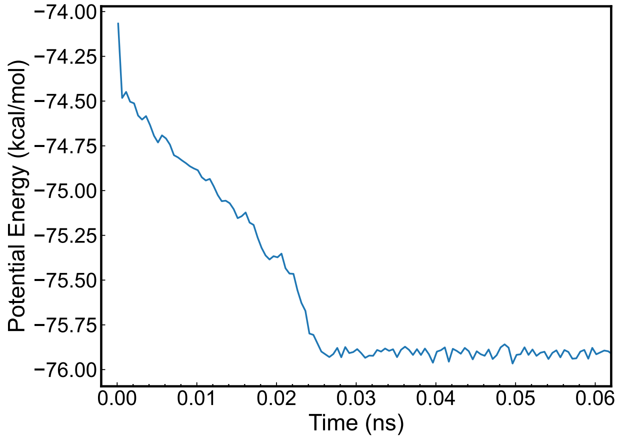

Figure S2: Potential energy as a function of time for the supercooled aluminum liquid-solid interface at 800 K. The system reaches equilibrium (full crystallization) at 0.03 ns.

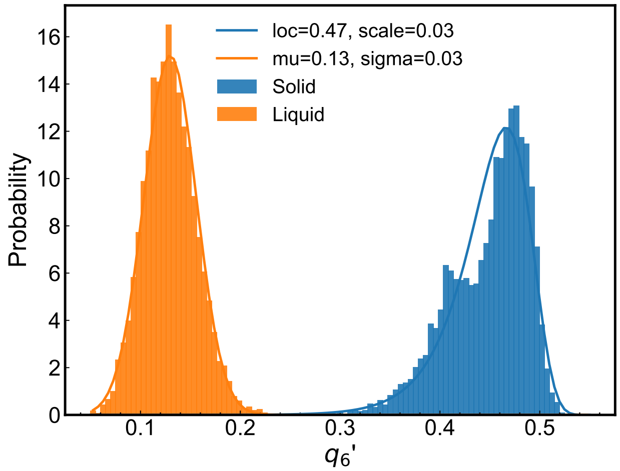

Figure S3: Histogram of the q_6' order parameter for bulk aluminum in solid and liquid states.

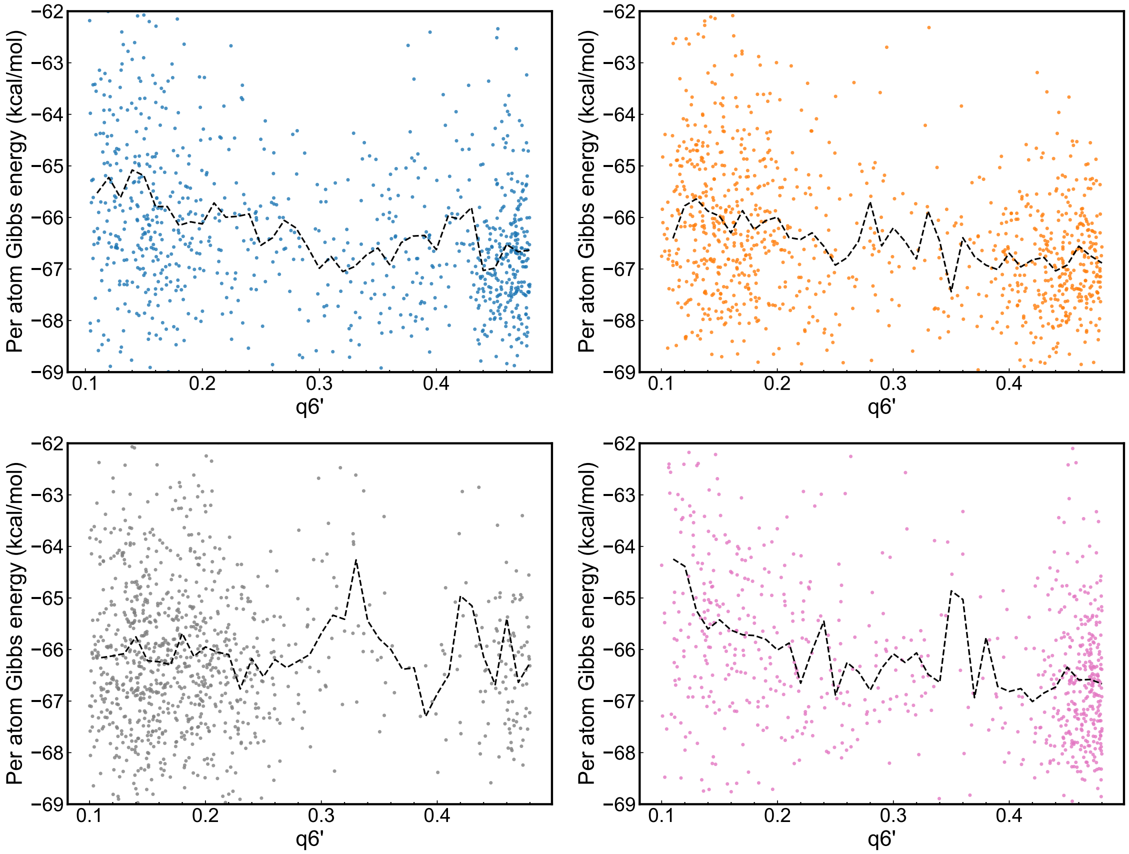

Figure S4: Gibbs free energy changes projected along the q_6' "reaction" coordinate of supercooled aluminum at 800 K and 1 atm. Typical pathways are shown with dashed lines representing the average, while the colors correspond to the data reported in Figure 3 of the main paper.

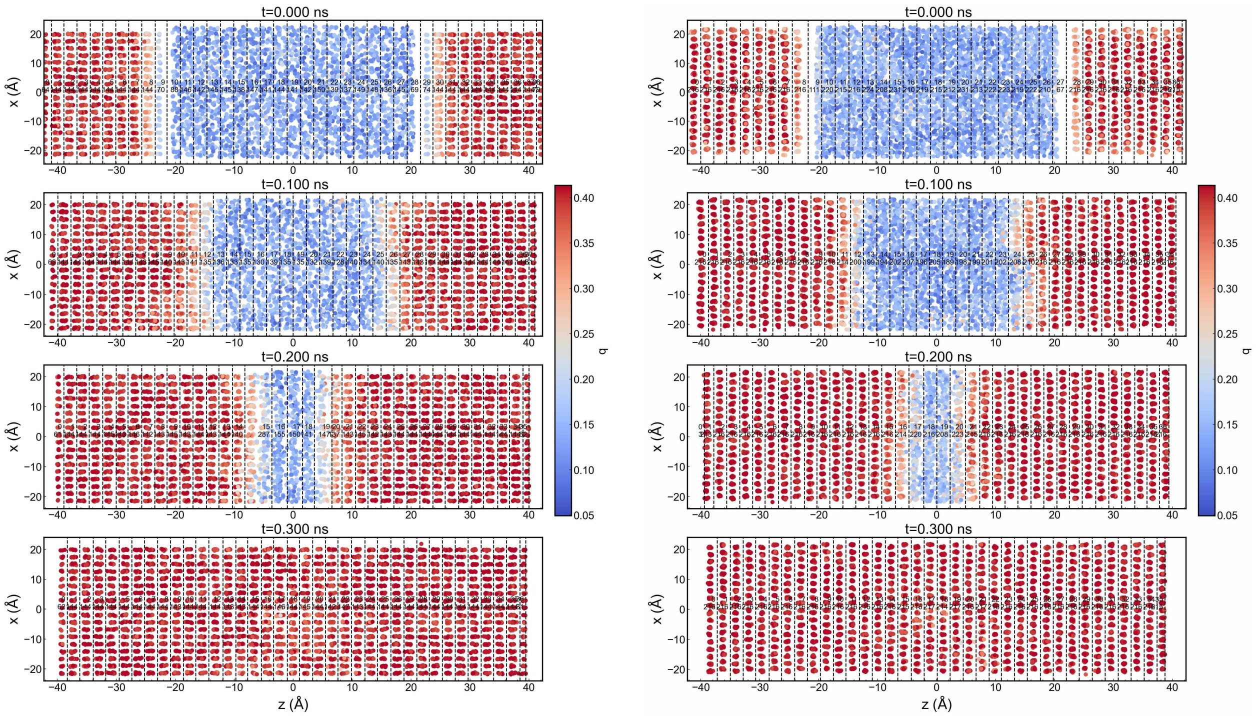

Aluminum atoms

Oxygen atoms

Figure S5: Simulation snapshots illustrating the crystal growth of supercooled alumina on the x-z plane and at 2000 K and 10 GPa. The left and right panels correspond to aluminum and oxygen atoms, respectively.

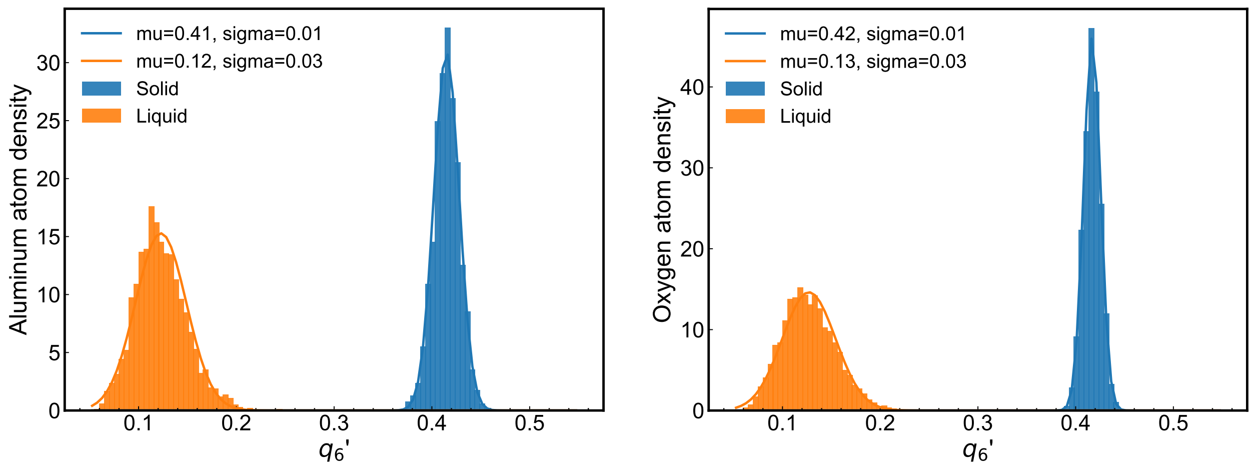

Aluminum atoms

Oxygen atoms

Figure S6: Histogram of the order parameter q_6' for bulk alumina in both solid and liquid phases. The results for aluminum atoms are displayed in the left panel, while those for oxygen atoms are shown in the right panel.

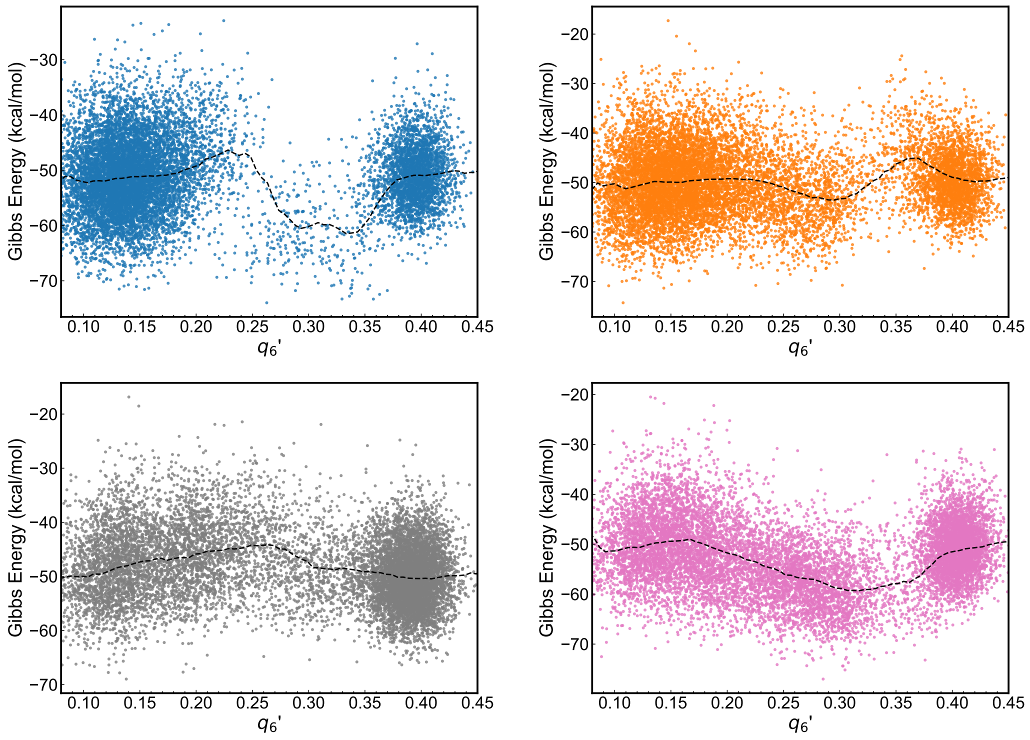

Figure S7: Gibbs energy changes projected along the “reaction” coordinate (order parameter q_6') for aluminum atoms in supercooled alumina at 2000 K and 10 GPa. The plots show typical free energy pathways for four clusters identified by the k-clustering method. See also Figure 5 in the main paper.

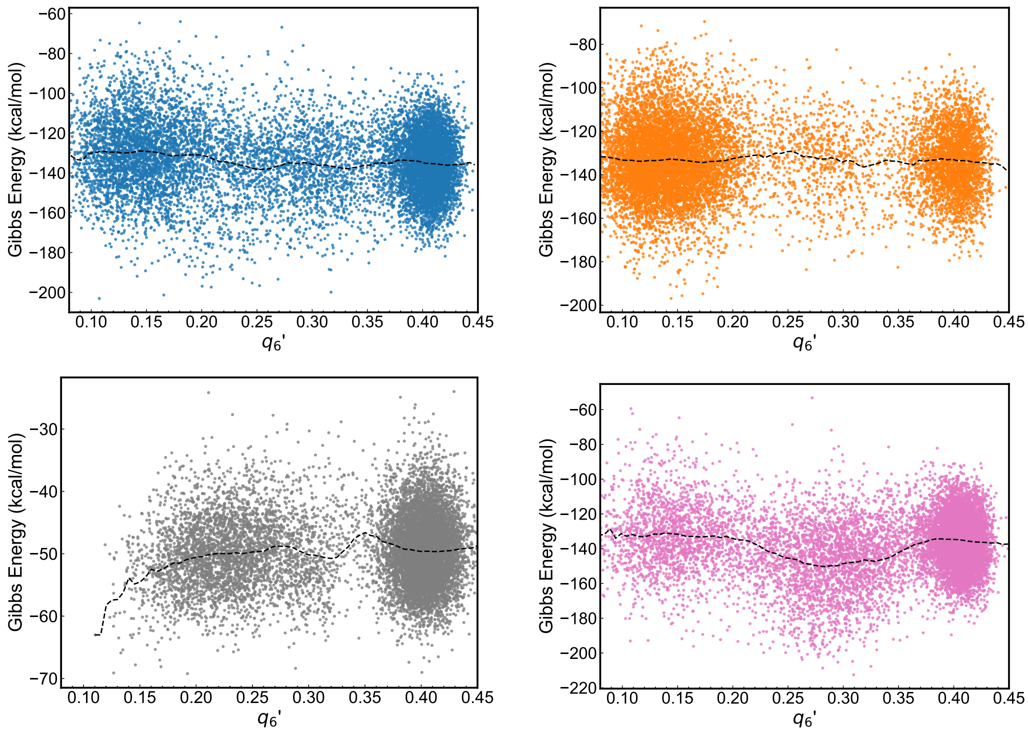

Figure S8: Same as Figure S7 for the oxygen atoms.

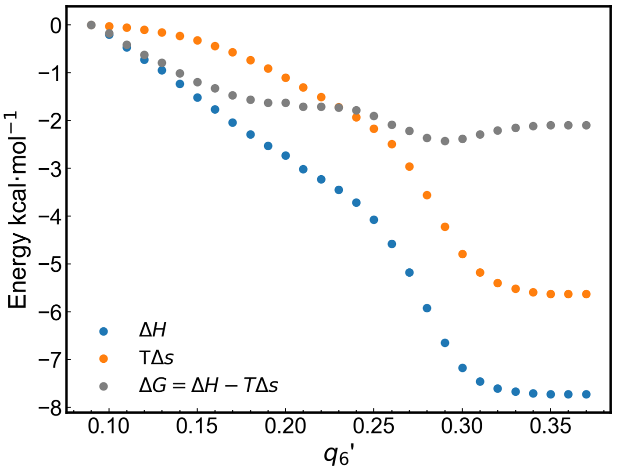

Figure S9: Enthalpy(ΔH), entropy (TΔs), and free energy (ΔG) for the crystal growth process in Silicon. The simulations were performed at 1 bar and 2000 K using the ReaxFF developed in reference[S7]

(S2) Hoover, W. G. Canonical dynamics: Equilibrium phase-space distributions. *Physical review A* **1985**, *31*, 1695.

(S3) Sun, J.; Liu, P.; Wang, M.; Liu, J. Molecular dynamics simulations of melting iron nanoparticles with/without defects using a reaxff reactive force field. *Scientific Reports* **2020**, *10*, 3408.

(S4) Zhao, H.; Bresme, F. Melting Point and Crystal Growth Kinetics of Metals and Metal Oxides Using Reactive Force Fields: The Case of Aluminum and Alumina. *Journal of Chemical Theory and Computation* **2024**, *20*, 8190–8201.

(S5) Thompson, A. P.; Aktulga, H. M.; Berger, R.; Bolintineanu, D. S.; Brown, W. M.; Crozier, P. S.; in 't Veld, P. J.; Kohlmeyer, A.; Moore, S. G.; Nguyen, T. D.; Shan, R.; Stevens, M. J.; Tranchida, J.; Trott, C.; Plimpton, S. J. LAMMPS - a flexible simulation tool for particle-based materials modeling at the atomic, meso, and continuum scales. *Comp. Phys. Comm.* **2022**, *271*, 108171.

(S6) Aktulga, H. M.; Fogarty, J. C.; Pandit, S. A.; Grama, A. Y. Parallel reactive molecular dynamics: Numerical methods and algorithmic techniques. *parallel computing* **2012**, *38*, 245–259.

(S7) Nayir, N.; Van Duin, A. C. T.; Erkoc, S. Development of the ReaxFF Reactive Force Field for Inherent Point Defects in the Si/Silica System. *The Journal of Physical Chemistry A* **2019**, *123*, 4303–4313.
